# Learning About the CS During Latent Inhibition: Preexposure Enhances Temporal Control

**DOI:** 10.1037/xan0000096

**Published:** 2016-02-15

**Authors:** Charlotte Bonardi, Ben Brilot, Dómhnall J. Jennings

**Affiliations:** 1 School of Psychology, University of Nottingham; 2 School of Biological Sciences, Plymouth University; 3 Institute of Neuroscience, Newcastle University

**Keywords:** association formation, Pavlovian conditioning, conditioned responding, latent inhibition, timing

## Abstract

In 3 experiments, rats were given nonreinforced preexposure to an auditory stimulus, after which this stimulus and a second, novel cue were paired with food. Lower rates of conditioned responding were observed to the preexposed stimulus across the 3 experiments, indicative of latent inhibition. The degree to which animals used these cues to time the occurrence of food delivery was also examined. Paradoxically, the response slopes—indicating the rate of increase in responding over the course of the conditioned stimulus—were greater for the preexposed than for the novel cues, consistent with the suggestion that the preexposed stimulus exerted greater temporal control. Moreover, this was the case irrespective of whether the duration of the cue during preexposure differed from that during conditioning. These results suggest that although conditioned stimulus preexposure retards conditioning, it may enhance timing. The findings are discussed in terms of current models of conditioning and timing.

Conditioning—learning that an environmental cue (conditioned stimulus, or CS) reliably signals an outcome (an unconditioned stimulus or US, often of motivational value)—is found across the animal kingdom. It is indicated by an elevation of *conditioned responding* during CS presentation, indicating increased anticipation of the US. However, other kinds of learning also occur during conditioning tasks. For example, animals learn to use the CS to *time* US occurrence: when the CS is of a fixed duration, subjects respond at increasingly high rates as the time of US delivery approaches (e.g., Kirkpatrick & Church, 2000); moreover, if the CS is presented for an extended period without reinforcement, there is a peak of responding at the point at which the US would normally occur, resulting in an inverted-U, Gaussian-type function (Roberts, 1981).

Although conditioning and timing have been shown to occur side-by-side in the same task (e.g., Balsam, Drew & Yang, 2002; Kirkpatrick & Church, 2000), the theoretical frameworks devised to explain these effects have tended to focus on one of the two phenomena (e.g., Gallistel & Gibbon, 2000; Jennings, Alonso, Mondragón, Franssen, & Bonardi, 2013; Kirkpatrick, 2014; Kirkpatrick & Church, 1998; Savastano & Miller, 1998). Theories of conditioning are often *associative*, attributing it to the formation of an association between the mental representations of the CS and the US, allowing presentation of the CS to activate the US representation and elicit a conditioned response. Such theories offer little explanation of timing effects (except for real-time models—see below). In contrast, some timing theories do offer an account of conditioning, but as a byproduct of the timing process (see Kirkpatrick, 2014 for a recent review). For example, hybrid time-based models assume that the CS is represented in a form that varies as a function of time^1^, which allows the time of reinforcement to be accurately encoded. In contrast, information-processing models assume that conditioning stems from sensitivity to temporal information in the conditioning episode. For example, scalar expectancy theory (SET; Gibbon & Balsam, 1981; cf. rate expectancy theory, Gallistel & Gibbon, 2000) assumes the animal computes the difference between the expected time to reinforcement in the *presence* of the CS, and in its absence; if the former value exceeds the latter by some criterion, the decision is made to respond (cf., Balsam & Gallistel, 2009; Kirkpatrick & Church, 2003).

Because of the theoretical gulf between associative and time-based accounts of conditioning, phenomena central to the associative perspective have been largely neglected by timing theories. One example is *latent inhibition*—that nonreinforced preexposure to a stimulus retards acquisition of conditioned responding to that stimulus, relative to a nonpreexposed cue (Lubow & Moore, 1959). Many associative theories interpret latent inhibition as a retardation in learning, resulting from a drop in the *associability* of the CS (e.g., Mackintosh, 1975; McLaren & Mackintosh, 2000; Pearce & Hall, 1980; Wagner, 1981). This may occur either as a function of the predictive ability of the CS (Mackintosh, 1975; Pearce & Hall, 1980), or the degree to which it is predicted, either by the context (Wagner, 1981), or by elements of the stimulus itself (cf. McLaren & Mackintosh, 2000). Others view it as a *performance* effect, produced by competition between a CS→nothing association formed during preexposure and the CS→US association established during conditioning (Bouton, 1993; Savastano, Yin, Barnet & Miller, 1998). Time-based accounts of conditioning, in contrast, say little about nonreinforced preexposure. Hybrid models typically focus on the temporal relation between specific states of the CS representation and *reinforcement*, while although information-processing theories assume information is gathered over a broad time window, which could include the preexposure phase (e.g., Balsam & Gallistel, 2009; Gallistel & Gibbon, 2000), it is unclear whether they predict latent inhibition. For example, according to SET, nonreinforced preexposure increases the expected time to reinforcement both in the presence of the CS *and* in its absence, leaving the ratio of these values unchanged relative to an untreated control group^2^ (see also rate expectancy theory; Gallistel & Gibbon, 2000).

Our aim was thus to examine the effect of stimulus preexposure on timing. While most associative theories do not account for timing explicitly, they could explain it by assuming that different portions of the CS condition to different degrees according to their proximity to the US; conditioning would therefore be at a maximum at the point of US delivery. This interpretation suggests that any factor retarding acquisition of conditioning will also retard acquisition of timing. Other associative theories have been formulated to incorporate timing directly; for example, the temporal coding hypothesis (Matzel, Held & Miller, 1988) proposes that associations incorporate information about the CS-US pairing—including the temporal relationship between them (e.g., Barnet, Grahame & Miller, 1993; Barnet, Cole & Miller, 1997; Blaisdell, Denniston & Miller, 1998). Although temporal coding does not explicitly address latent inhibition, if stimulus preexposure were to produce an association between the CS and no outcome, it would predict that the duration of the stimulus would also be encoded. When the CS is then paired with the US, potential for competition between these two associations arises (e.g., Bouton, 1993), which these authors have argued is the source of latent inhibition (cf. Savastano et al., 1998). Temporal coding thus predicts that this interference, and hence latent inhibition, will be greater when the duration of the CS during preexposure and conditioning match—and the same might also apply to timing ability.

In contrast, time-based theories of conditioning make no clear predictions. While hybrid models say little about nonreinforced exposure, information processing models like SET predict that timing occurs only *after* conditioned responding has been acquired (although see, e.g., Balsam, Drew & Yang, 2002; Kirkpatrick & Church, 2000). Thus, *if* they could predict latent inhibition they should also predict retardation of timing—but it is not clear they can predict latent inhibition in the first place.

In summary, the temporal coding hypothesis predicts that timing will be retarded by preexposure, and that this effect will be maximal when the duration of the stimulus during preexposure matches that during conditioning. Associative theories interpreting latent inhibition as an effect on learning may also predict it will retard timing (e.g., Mackintosh, 1975; McLaren & Mackintosh, 2000; Pearce & Hall, 1980; Wagner, 1981), although (compared to temporal coding) the boundary conditions of this are unspecified. Finally, time-based theories of conditioning make no clear predictions about the effect of preexposure on either conditioning or timing effects.

## Experiment 1

In the first experiment two groups of rats were preexposed to an auditory CS, after which this and a second, novel cue were each reinforced after 40 s with two food pellets. We anticipated preexposure would result in latent inhibition—slower learning to the preexposed than to the novel CS. The groups’ treatment differed in the preexposure phase. Although both groups received the same total duration of CS exposure, for Group Same CS presentations were always of a fixed, 40-s duration, exactly as during conditioning, whereas for Group Different the preexposed CS varied in duration from trial to trial according to a uniform distribution with an average of 40 s), such that it was either longer or shorter than, but never equal to, 40 s. Thus Group Different—unlike Group Same —did not have any experience of the conditioning duration (see Table 1).[Table-anchor tbl1]

In both groups we anticipated seeing latent inhibition—slower acquisition of conditioned responding to the preexposed CS—and we expected it to be similar in magnitude because total CS exposure was equated: Ayres, Philbin, Cassidy, Bellino, and Redlinger (1992) found this factor was the most important determinant of the degree of latent inhibition, and that matching the CS duration during preexposure and conditioning had little effect (contrary to the predictions of temporal coding). But the key issue was whether preexposure would also impair learning to *time* the duration of the preexposed CS—and if so, whether this would depend on the temporal properties of preexposure. If timing is impaired *whatever* the duration of the CS during preexposure, then timing the preexposed cue should be similarly affected in both groups; but if it depends on preexposure to the *conditioning duration* (as temporal coding predicts), then timing should be worse in Group Same, but *not* in Group Different. To assess timing we computed the rate of conditioned responding in each 1-s bin of the CS, and computed the *slope* of this function. Timing would be evident as an increase in rate of conditioned responding as US delivery approaches, resulting in positive slopes. In contrast, if there is no timing (as is found, e.g., early in training or when the stimulus is of variable duration), response rates are steady over the course of the CS, and slopes are close to zero (e.g., Jennings et al., 2013).^3^ As we were interested in the effect of latent inhibition on timing, it was critical that we could demonstrate a reliable latent inhibition effect. Although preexposure retards conditioning, it typically does not reduce asymptotic levels of responding, meaning latent inhibition is a transient phenomenon that dissipates with extended testing. For this reason we restricted the number of training sessions, so that asymptotic levels of responding had not been reached. However, orderly response slopes do not develop immediately, but require a certain amount of training to emerge. Thus we tested the animals at around the point at which responding was just about to asymptote, which in the experiments that follow was between 4 to 6 training sessions.

### Method

#### Subjects

Subjects were 16 male Lister hooded rats (Charles River, United Kingdom) with a mean free-feeding weight of 330 g (range: 305–350 g). The rats were weighed daily and their daily food ration restricted such that they were gradually reduced to 85% of their free-feeding weights before the start of the experiment. They were maintained at this level throughout training, their target 85% level being adjusted weekly according to a growth curve so that their target weights increased gradually over the course of the experiment. Water was freely available in the home cages. They were maintained on a 12-hr light/dark cycle, with the lights turned on at 7am, and temperature was maintained at 21 °C (±1); the humidity was 60% (±10%). There were eight animals per group.

#### Apparatus

All conditioning and testing procedures were conducted in 8 identical chambers (20 × 24 × 30 cm), each of which was situated in a ventilated, noise-attenuating shell (74 × 38 × 60 cm; MED Associates). Each chamber was equipped with a speaker for delivering auditory stimuli, a houselight, a foodcup and two jewel lights, one situated on each side of the food cup. The houselight was not employed. A speaker, located on the right side of the back wall of the chamber on the opposite wall from the food cup, could deliver two 70-dB auditory stimuli (scale A, measured near the food hopper), a white noise and a 10-Hz click. A pellet dispenser (Model ENV-203) delivered 45-mg Noyes (Improved Formula A) pellets into the food cup. Each head entry into the food cup was recorded by breaking an infrared photobeam and recorded as a response. Med-PC for Windows (Tatham & Zurn, 1989), running on a PC, controlled experimental events.

#### Procedure

##### Preexposure

All subjects received 10 sessions of preexposure, each comprising 42 presentations of Stimulus A (which for half the subjects in each group was the noise, and for the remainder the click). For subjects in Group Same presentations of A had a fixed duration of 40 s on every trial. For subjects in Group Different the 42 presentations of A were of variable durations drawn without replacement from a uniform distribution, with a mean duration of 40 s: Thus, each block of six trials comprised CS presentations of the following durations: 20, 28, 36, 44, 52, and 60 s, and there were seven blocks per session. The intertrial interval (ITI) in this and the conditioning phase comprised a fixed interval of 130 s plus a variable interval drawn from an exponential distribution with a mean of 60 s (i.e., 130 + ∼60 s).

##### Conditioning

During this stage all subjects received 40 trials per session: 20 of these presentations were with CS A, to which they had been preexposed, and 20 to the novel B. For animals for which A had been the click, B was the noise, and vice versa. All CS presentations were of 40-s duration, and were preceded by a 40-s pre-CS period, which was a portion of the ITI during which responding was also recorded. Each CS presentation was immediately followed by the delivery of two food pellets, and the two trial types were intermixed in a semirandom order. There were five sessions in this stage.

#### Data treatment

Mean response rates during each type of trial were obtained by computing the total number of responses made during each CS type in each session, and during the corresponding pre-CS periods, and converting to responses per minute (rpm). Conditioned responding in each session was indexed by a *difference* score—the mean response rate during each type of CS after subtraction of the rate during the corresponding pre-CS periods. This provided a measure of the degree to which CS presentation elevated conditioned responding over background levels.

#### Timing

The total number of responses occurring in each successive 1-s time bin of each type of CS, pooled over all the conditioning sessions, was computed, and the rate of responding in each bin calculated for each rat. The resultant distributions were smoothed by taking an average over successive 5-s bins (i.e., Bins 1–5, 2–6, etc.) to reduce the influence of transient variation in responding (e.g., Church, Meck, & Gibbon, 1994; Tam, Jennings, & Bonardi, 2015; Matell, Kim, & Hartshorne, 2014). These rates were then normalized (divided by the total number of responses for that rat) to give the percentage of responses in each 1-s time bin, to ensure each animal contributed equally to the shape of the functions regardless of its response rate. Then a linear function was fitted to each normalized response function, and the slope determined from the best-fitting linear curve for each rat (linear fits provide a good characterization of response rates over CS duration: Jennings, Bonardi, & Kirkpatrick, 2007; cf. Kirkpatrick & Church, 2000). A higher slope score, relative to a low or negative score, indicates that a greater proportion of an individual’s head-entry responses are distributed toward the end of the CS, consistent with temporally accurate anticipation of US delivery; thus, slope was used as an estimate of temporal control (Kirkpatrick & Church, 2000; Jennings & Kirkpatrick, 2006).

Data were analyzed using analysis of variance; significant interactions were explored with simple main effects analysis, using the pooled error term. Partial eta squared (η_p_^2^) and its 95% confidence interval (CI) were given for significant effects and interactions in the analyses of variance (ANOVAs).

### Results

No data were collected during the preexposure stage.

#### Conditioning

**Figure 1 fig1:**
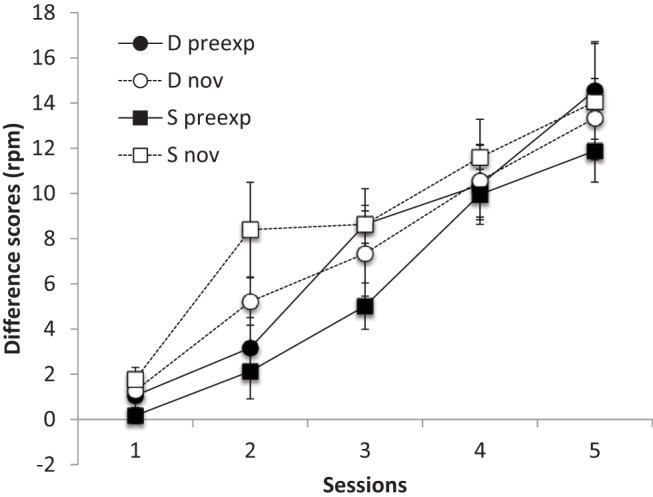
Group mean difference scores (response rate during CS – response rate during pre-CS period) for the preexposed (preexp) and novel (nov) CSs in each of the five conditioning sessions (11–15) of Experiment 1. Error bars show standard error of the mean.

The results of the conditioning phase are shown in Figure 1. Rates of responding to the preexposed cue appeared lower than to the novel stimulus in Group Same, indicating latent inhibition; this was less evident in Group Different. ANOVA with preexposure (preexposed or novel), session and group (Same or Different) as factors revealed a significant main effect of session *F*(4, 56) = 54.33, *p* < .001, *MSe* = 13.89, η_p_^2^ = .79, 95% CI [.67, .84], which interacted with preexposure *F*(4, 56) = 3.01, *p* < .026, *MSe* = 5.94, η_p_^2^ = .18, 95% CI [.00, .30]; nothing else was significant, largest *F*(1, 14) = 3.89, *p* = .069, *MSe* = 24.29, η_p_^2^ = .22, for the Preexposure × Group interaction. Further analysis of the Preexposure × Session interaction revealed a significant effect of preexposure on Session 2, *F*(1, 70) = 14.43, *p* < .001, *MSe* = 9.61, but on no other session, largest *F*(1, 70) = 1.12, *p* = .293, *MSe* = 9.61, for Session 3.[Fig-anchor fig1]

**Figure 2 fig2:**
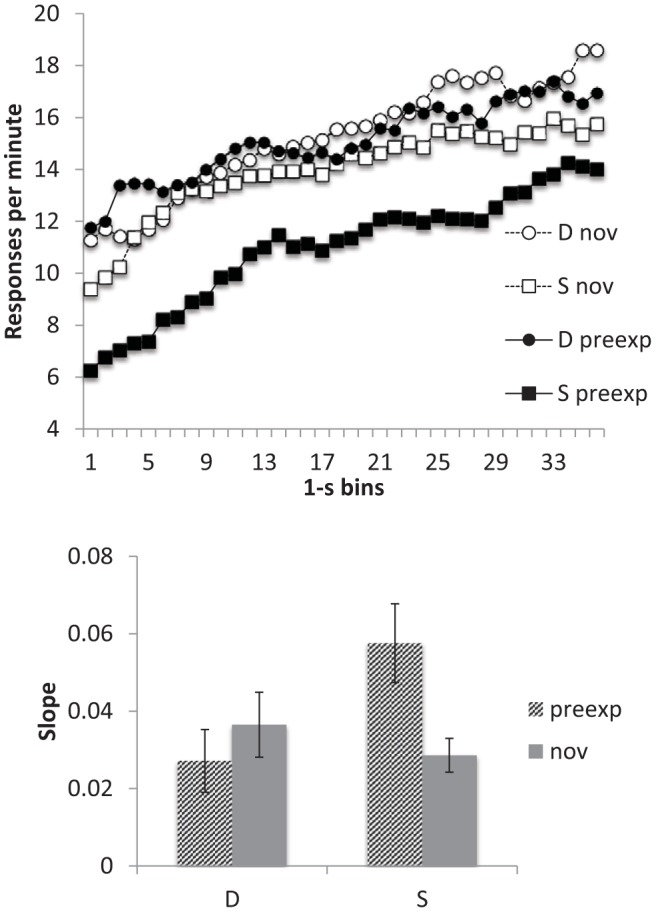
Group mean scores for Group Same (S) and Different (D) for the preexposed (preexp) and novel (nov) CSs from the conditioning sessions (11–15) of Experiment 1. Upper panel: Smoothed response rates in each 1-s bin. Lower panel: Slopes. Error bars represent standard error of the mean.

The Preexposure × Group interaction, although not significant (*p* = .069) is consistent with latent inhibition being weaker in Group Different, as is suggested in Figure 2; indeed after four sessions the Preexposure × Group interaction was significant, *p* = .039, and a significant latent inhibition effect was found in Group Same, *F*(1, 14) = 7.72, *p* = .015, *MSe* = 5.66, η_p_^2^ = .47, but not in Group Different, *F* < 1. Thus there is evidence that latent inhibition was weaker in Group Different, at least after four sessions.[Fig-anchor fig2]

The mean rate of pre-CS responding in each session was 4.36, 6.43, 4.50, 3.70, and 2.84 rpm for Group Same, and 6.10, 9.60, 7.33, 5.80, and 5.49 for Group Different. ANOVA with group and sessions as factors revealed only a significant effect of sessions, *F*(4, 56) = 12.56, *p* < .001, *MSe* = 5.66, η_p_^2^ = .47, 95% CI [.24, .58]; nothing else was significant, largest *F*(1, 14) = 3.38, *p* = .09, *MSe* = 73.44, for the main effect of group.

#### Timing

The smoothed response rates, averaged over all five sessions, are shown in the top panel of Figure 2. Response rates appeared lower to the preexposed than to the novel cue in Group Same, but this difference was not apparent in Group Different. This reflects the pattern seen in Figure 1 and again suggests a more marked latent inhibition effect in Group Same than in Group Different. There was also some suggestion that the difference in response rate between the start and the end of the CS was greater for the preexposed cue in Group Same than in the other conditions. This pattern was evident in the averaged slope data (lower panel of Figure 2), where it may be seen that the mean slope for the preexposed CS in Group Same appeared higher than for the other conditions, which did not seem to differ substantially. ANOVA with CS and group as factors confirmed that there was a significant interaction between these two factors, *F*(1, 14) = 6.62, *p* = .022, *MSe* = .0001, η_p_^2^ = .32, 95% CI [.00, .58], and simple main effects tests revealed a difference between preexposed and novel CSs in Group Same, *F*(1, 14) = 7.57, *p* = .016, *MSe* = .0001, but not in Group Different, *F* < 1.

### Discussion

We anticipated equal latent inhibition in both groups; yet, although statistically this was the case after five sessions, the effect in Group Different was numerically weak, and after four sessions latent inhibition was actually present only in Group Same. There are reasons to expect such a difference. It is well established that latent inhibition is context-specific: if a stimulus is preexposed and conditioned in distinctly different contexts, then latent inhibition is attenuated, compared when the context of preexposure and conditioning is the same (Channell & Hall, 1983). If in Group Different the change between experiencing many stimulus durations during the preexposure phase to just one during conditioning constituted such a context change, this would have reduced the magnitude of latent inhibition observed.

But there are alternative explanations. First, as we saw above, the temporal coding hypothesis predicts greater latent inhibition when the durations of the preexposed and conditioned CSs match (Matzel et al., 1988). Although previous studies have reported that this factor has little effect on latent inhibition (Ayres et al., 1992), they used only *fixed* duration stimuli, and so the result we observed could be related to the fact that the preexposed cue in Group Different was variable.

There are other ways using a variable CS could influence latent inhibition. For example, according to the Pearce & Hall (1980) model, latent inhibition results from a drop in associability of stimuli that are reliably followed by no consequence. Although this model makes no assumptions about the temporal structure of a stimulus, one common associative approach is to conceptualize each CS as a set of shorter elements, corresponding to successive time bins that are activated in a fixed sequence after CS onset (e.g., Konorski, 1948; Wagner & Rescorla, 1972; cf. Pavlov, 1927; Vogel, Castro, & Saavedra, 2003). In a CS that is always of the same duration, the first element will reliably predict the second, which will reliably predict the third and so on. Thus, each element is being reinforced by the next according to a continuous reinforcement schedule; hence, while the associability of the final element will fall because it reliably predicts no consequence, that of preceding elements could also fall because each reliably predicts the element that follows it. In contrast, if CS duration varies from trial to trial, each element is no longer reliably followed by the next—and this will result in the individual stimulus elements maintaining their associability, which could therefore reduce or eliminate the latent inhibition effect. An attempt to discriminate between these possibilities was the aim of Experiment 2.

The second notable finding was the effect of preexposure on timing. Contrary to our expectation, no impairment in temporal control was observed—rather, the mean slope of the preexposed CS was greater in Group Same. Thus, the group that showed the most robust latent inhibition effect also displayed greater temporal control after preexposure in these animals. Experiment 2 also aimed to replicate this finding.

## Experiment 2

Two groups of rats, Group Same and Group Different, were preexposed to *two* stimuli, A and a control stimulus C (see Table 1). For Group Same, A was preexposed at a fixed 20-s duration, and C with a variable duration that was either longer or shorter but never equal to 20 s. Group Different received the converse arrangement, A being variable and C fixed. Then both groups were conditioned to A, and also to a novel cue B, both with a fixed 20-s duration. We reduced the mean CS duration from 40 s to 20 s to equate the total amount of stimulus exposure with that given in Experiment 1. Although this also halved the amount of preexposure given to the Target Stimulus A, previous work has shown that this is more than enough required to give a robust latent inhibition effect.

As all animals experienced stimuli of both variable *and* fixed durations during preexposure, this should equate the similarity of the preexposure and conditioning contexts more closely than in Experiment 1. Thus, if the apparent failure to observe robust latent inhibition in Group Different of Experiment 1 was due to a context change for Group Different but not Group Same, then latent inhibition should be equally strong in both groups in the present study. But if it was due to the change in the temporal nature of the to-be-conditioned stimulus between preexposure and test (cf. Matzel et al., 1988), or the ability of variable stimulus presentations to maintain associability during preexposure (cf. Pearce & Hall, 1980), then latent inhibition should be greater in Group Same than in Group Different, just as in the previous experiment.

### Method

#### Subjects and apparatus

Subjects were 16 male Lister hooded rats (Charles River, United Kingdom) with a mean free-feeding weight of 352 g (range: 329–372 g). They were housed and food restricted exactly as in Experiment 1. The apparatus was identical to that of Experiment 1, except for the addition of a third auditory stimulus, a 75-db 4-kHz tone.

#### Procedure

##### Preexposure

Subjects received 10 sessions of preexposure, each comprising 42 presentations of A and 42 of C. A and B were counterbalanced between the click and the noise, as in Experiment 1, and C was always the tone. The trials were arranged in 12-trial blocks, each comprising 6 presentations of A and 6 of C. For subjects in Group Same presentations of A had a fixed duration of 20 s on every trial, while C’s duration was drawn without replacement from one of the following durations: 10, 14, 18, 22, 26, and 30 s. For Group Different, these treatments of A and C were reversed. The ITI in this and the conditioning stage comprised a fixed interval of 40 s and a variable interval drawn from an exponential distribution with a mean of 40 s (40 + ∼40 s).

##### Conditioning

The five conditioning sessions each comprised 20 trials with A and 20 with B, intermixed in a semirandom order. Both stimuli were presented at a fixed 20-s duration, and followed by the delivery of two food pellets; the 20-s period of the ITI immediately preceding each CS presentation constituted the pre-CS period.

#### Data treatment

This was identical to that in the previous experiment.

### Results

#### Conditioning

**Figure 3 fig3:**
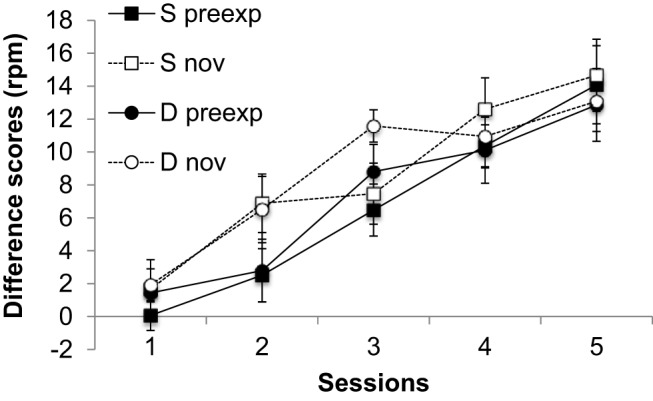
Group mean difference scores (response rate during CS – response rate during pre-CS period) for the preexposed (preexp) and novel (nov) CSs in each of the five conditioning sessions (11–15) of Experiment 2. Error bars represent standard error of the mean.

The results of the conditioning phase, summarized in Figure 3, suggest that latent inhibition was present in both groups; ANOVA with preexposure (preexposed or not), session and group (Same or Different) as factors revealed a significant main effect of preexposure *F*(1, 14) = 7.54, *p* = .016, *MSe* = 16.84, η_p_^2^ = .35, 95% CI [.01, .60], and also of session *F*(4, 56) = 39.91 *p* < .001, *MSe* = 19.58, η_p_^2^ = .74, 95% CI [.59, .80]; nothing else was significant, largest *F*(4, 56) = 1.37, *p* = .255, *MSe* = 19.58.[Fig-anchor fig3]

The mean rates of pre-CS responding in each sessions were 5.00, 8.63, 7.28, 6.10 and 4.86 rpm for Group Same, and 4.42, 11.44, 5.63, 7.76, and 4.98 for Group Different. ANOVA with group and sessions as factors revealed a significant main effect of sessions, *F*(4, 56) = 12.45, *p* < .001, *MSe* = 11.78, η_p_^2^ = .47, 95% CI [.24, .58]; nothing else was significant, largest *F*(4, 56) = 2.15, *p* = .087, *MSe* = 11.78 for the Session × Group interaction.

#### Timing

**Figure 4 fig4:**
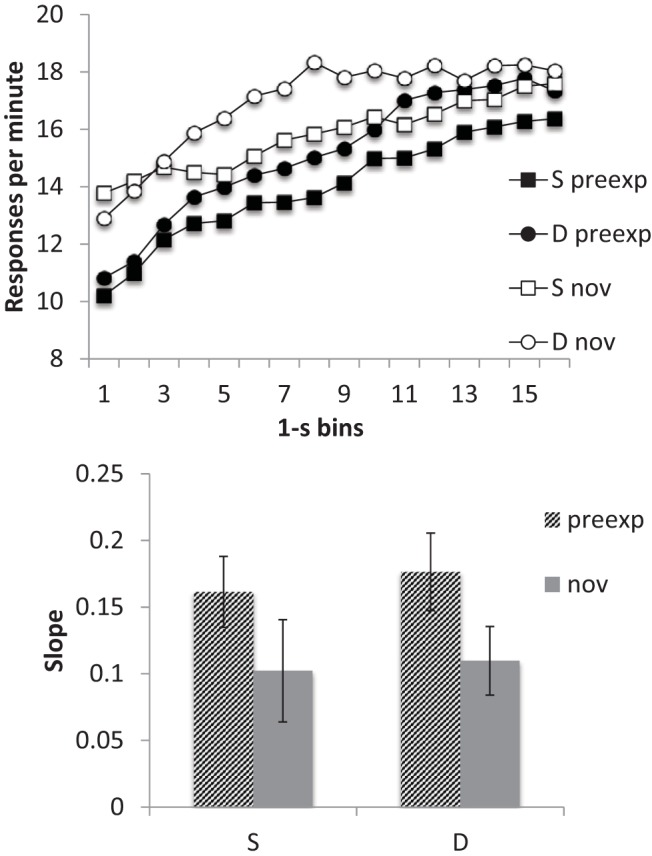
Group mean scores for Group Same (S) and Different (D) for the preexposed (preexp) and novel (nov) CSs from the conditioning sessions (11–15) of Experiment 2. Upper panel: Smoothed response rates in each 1-s bin of the preexposed (preexp) and novel (nov) CSs. Lower panel: Slopes. Error bars represent standard error of the mean.

The smoothed response rates, averaged over all five sessions, are shown in the upper panel of Figure 4. Responding at the start of the preexposed stimuli appeared to be lower than to the novel cues, an effect that dissipated as the CS continued; this is consistent with the preexposed stimulus having steeper slope functions than those for the novel cues.[Fig-anchor fig4]

A similar conclusion was suggested by the averaged slope data (Figure 4 lower panel): slopes were higher for preexposed than for novel cues in both groups. ANOVA with preexposure and group as factors revealed a significant main effect of preexposure, *F*(1, 14) = 4.92, *p* = .044, *MSe* = .006, η_p_^2^ = .26, 95% CI [.00, .54]; nothing else was significant, *F*s < 1. Thus, the response slopes were greater following the preexposure treatment consistent with a higher level of temporal control.

### Discussion

Just as in Experiment 1, Group Same was conditioned to a stimulus duration which they had experienced during preexposure to that CS, while Group Different was not. In this study, unlike in Experiment 1, robust latent inhibition—lower rates of conditioned responding to the preexposed cue—was evident in Group Different. The main difference was that in the present experiment *both* groups had experience of variable duration stimuli during preexposure, which aimed to equate preexposure and conditioning contexts more closely for Group Different than was the case in Experiment 1. The fact that minimizing the likelihood of context-specificity in Group Different allowed the emergence of strong latent inhibition suggests that this was the factor reducing the size of this effect in Experiment 1, rather than because preexposure to a variable stimulus maintains its associability (Pearce & Hall, 1980), or because a match between CS durations in preexposure and conditioning maximizes latent inhibition (Matzel et al., 1988). That the degree of latent inhibition observed is little affected by a mismatch in stimulus duration between preexposure and conditioning is consistent with previous findings by Ayres et al. (1992).

The present experiment also replicated the finding from Experiment 1 that the mean slope of the response distributions was greater for the preexposed than for the novel cues—and this was true in both groups. This result is not consistent with the predictions of the conditioning theories outlined above, none of which predicted the enhancement in temporal control that such a difference seems to reflect. In Experiment 1 this effect was evident in Group Same although not in Group Different—despite the fact that both underwent stimulus preexposure. But in Experiment 1 latent inhibition appeared markedly smaller in Group Different, an effect that was supported by analysis of the data after four sessions. The suggestion is that the enhancement in the slope of the response function might be a byproduct of latent inhibition itself.

We argued above that if learning to time depended on successful association formation, then theories viewing latent inhibition as a retardation in association formation (e.g., Pearce & Hall, 1980; Mackintosh, 1975; McLaren & Mackintosh, 2000; Wagner, 1981) might also predict a deficit in timing. But this analysis, although perhaps plausible at face value, is possibly naïve. Associative theories often consider the CS as being composed, at least in part, of constituent elements that are activated in a consistent temporal sequence (Vogel et al., 2004). If early portions of the CS condition less effectively than later ones, this would result in increasing levels of conditioned responding as the US approaches, and produce the characteristic timing function. The steeper slope observed in the preexposed CS could, then, be interpreted in terms of differential latent inhibition accruing to the various portions of the CS. If more latent inhibition were suffered by initial portions of the CS than by later ones, this could selectively retard conditioning at the start of the CS, and produce the pattern of results we observed; discussion of potential mechanisms for this will be taken up below. Alternatively, the effects of latent inhibition might be less evident when levels of responding are lower—such as at the *start* of the CS, which will condition less well because the US is temporally distant. Both suggestions require us to assume that conditioning, and perhaps also latent inhibition, can occur relatively independently to earlier and later portions of the CS.

One aspect of our results could help us to evaluate this suggestion. In Group Same, all temporal elements of the CS would have been preexposed on the same number of occasions, as the entire stimulus was present on every preexposure trial. In contrast, for subjects in Group Different, preexposure trials with the target stimulus were either longer or shorter than the to-be-conditioned stimulus, so that while earlier elements of the CS were present on every preexposure trial, later ones were not. If the degree of latent inhibition to a CS element is determined by the amount of preexposure it receives, then these earlier portions should have conditioned less well than the later ones, accentuating the slope of the response function. This analysis predicts that the slope for the preexposed cue should have been greater in Group Different than in Group Same.

Although there was no evidence of this, it may be that the previous experiments were insufficiently sensitive to detect such a difference. The discrepancy in preexposure between the start and the end of the stimulus in Group Different was not that great—the first quarter of the CS was present on 100% of the preexposure trials, and the final quarter on approximately 67%, a difference of only 33%. Moreover, it is possible that latent inhibition produced by this amount of preexposure was close to ceiling, making differences difficult to detect. The aim of the third experiment was to replicate the effect of preexposure on timing, and at the same time enhance the chances of observing the contribution of such a mechanism.

## Experiment 3

### Experiment 3a

Experiment 3a was a replication of Experiment 2, but with some modifications (see Table 1). First, preexposure was altered to increase the difference in exposure to the initial and final portions of the CS. Two groups of rats, Group Same and Group Different, were both preexposed to A and C. For Group Same, A was always of 20-s duration, whereas C was 10 s on half the occasions it was presented, and 30 s on the remainder. Group Different experienced the converse arrangement. Then, as in Experiment 2, both groups were conditioned to A and a novel stimulus B, both of which were followed by food after 20 s.

For Group Different the first quarter of the 20-s CS was present on 100% of the preexposure trials, and the final quarter on 50%, giving a discrepancy of 50%—greater than the 33% differential in Experiment 2. In addition, the total amount of preexposure was halved from that in Experiment 2; this aimed to reduce the possibility that latent inhibition was close to ceiling, potentially masking any differences that might be present.

#### Method

##### Subjects and apparatus

Subjects were 16 male Lister hooded rats (Charles River, United Kingdom) with a mean free-feeding weight of 296 g (range: 276–310 g). They were housed and food-restricted exactly as in Experiment 1. The apparatus was identical to that of Experiment 1.

##### Procedure

###### Preexposure

All subjects received 10 sessions of preexposure, each comprising 20 presentations of stimulus A, and 20 of stimulus C, intermixed in a semirandom order. For subjects in Group Same presentations of A had a fixed duration of 20 s on every trial, while C’s duration was 10 s on half the occasions on which it was presented, and 30 s on the remainder. For subjects in Group Different these arrangements were reversed (see Table 1). In all other respects this stage was identical to that of the previous experiment.

###### Conditioning

Conditioning sessions were identical to those of the previous experiment. There were six sessions in this stage.

##### Data treatment

In the conditioning stage one rat from Group Same was identified as an outlier, responding substantially more to the preexposed cue than to the novel cue,^4^ and so was omitted from all subsequent analyses. Moreover, possibly as a result of the deliberately reduced number of preexposure trials, latent inhibition was less robust in the present experiment, only being evident after six sessions; thus the data from six rather than five conditioning sessions are considered in the analyses below. In all other respects data analysis was identical to that of the previous experiment.

#### Results

##### Conditioning

**Figure 5 fig5:**
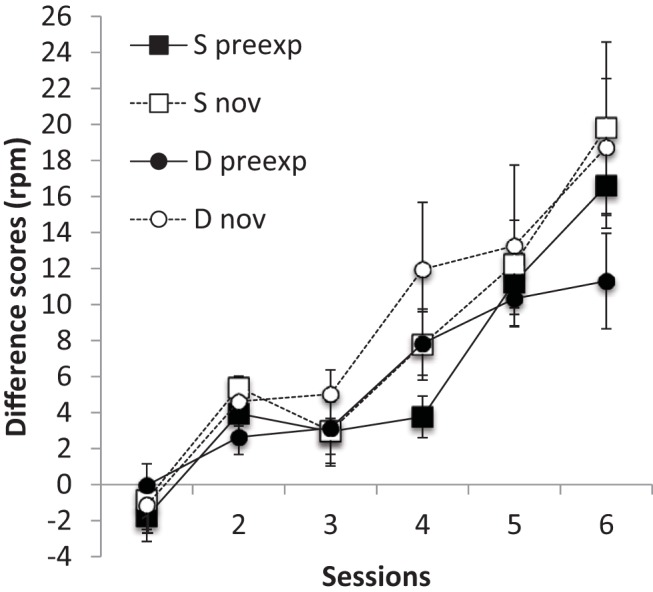
Group mean difference scores (response rate during CS – response rate during pre-CS period) for the preexposed (preexp) and novel (nov) CSs in each of the six conditioning sessions (11–16) of Experiment 3a. Error bars represent standard error of the mean.

The results of the conditioning phase are shown in Figure 5. Latent inhibition was again apparent in both groups: ANOVA with preexposure (preexposed or not), session and group (Same or Different) as factors revealed a significant effect of preexposure *F*(1, 13) = 6.41, *p* = .025, *MSe* = 36.27, η_p_^2^ = .33, 95% CI [.00, .59], and also of session, *F*(5, 65) = 26.13 *p* < .001, *MSe* = 45.61, η_p_^2^ = .67, 95% CI [.50, .73]; nothing else was significant, largest *F*(5, 65) = 1.83, *p* = .120, *MSe* = 15.38.[Fig-anchor fig5]

The mean rates of pre-CS responding in each sessions were 9.60, 6.36, 12.98, 9.84, 7.35, and 8.65 rpm for Group S, and 10.94, 5.57, 12.10, 11.22, 11.59, and 8.87 for Group D. ANOVA with group and sessions as factors revealed only a significant effect of sessions, *F*(5, 65) = 5.64, *p* < .001, *MSe* = 25.31, η_p_^2^ = .30, 95% CI [.09, .42]; nothing else was significant, largest *F*(5, 65) = 1.06, *p* = .388, *MSe* = 25.21 for the Session × Group interaction.

##### Timing

**Figure 6 fig6:**
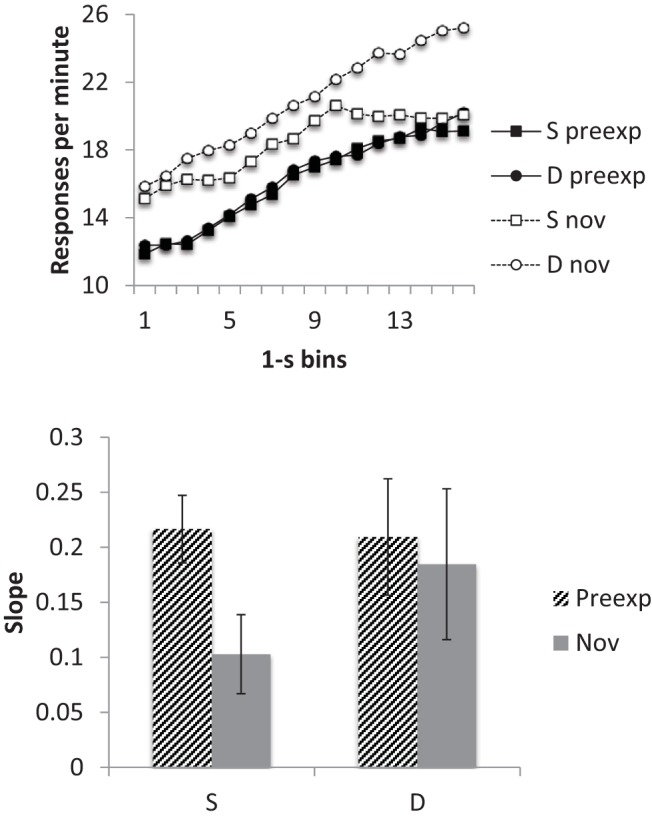
Group mean scores for Group Same (S) and Different (D) for the preexposed (preexp) and novel (nov) CSs from the conditioning sessions (11–16) of Experiment 3a. Upper panel: Smoothed response rates in each 1-s bin. Lower panel: Slopes. Error bars represent standard error of the mean.

The smoothed response rates, averaged over all six sessions, are shown in the upper panel of Figure 6. In Group Same responding was lower to the preexposed than to the novel cue at the start of the CS, but this difference dissipated over the course of the stimulus; in contrast, in Group Different responding appeared to be lower to the preexposed cue throughout the entire stimulus. Moreover, any differences between the two groups were in responding to the novel CS—the timing functions for the preexposed CSs were approximately superimposed. The averaged slope data are presented in the lower panel of Figure 6; slopes were again higher for the preexposed cues, evidenced by a significant effect of preexposure, *F*(1, 13) = 5.25, *p* = .039, *MSe* = .007, η_p_^2^ = .29, 95% CI [.00, .56]; although this effect seemed clearer in Group Same, the Group × Preexposure interaction was not significant *F*(1, 13) = 2.17, *p* = .164, *MSe* = .007. Nothing else was significant, *F* < 1.[Fig-anchor fig6]

#### Discussion

In this experiment latent inhibition was again similar in the two groups. We also replicated the effect on temporal control shown in the previous experiments: Response function slopes were significantly greater for the preexposed cues in both groups of animals.

Nonetheless there was no evidence that this enhancement in slope was more pronounced in Group Different; if anything the effect was weaker in these animals. Thus Experiment 3b tried a more extreme strategy to facilitate observation of this effect. It may be that *any* preexposure to a portion of the CS produces enough latent inhibition to make differences in conditioning difficult to observe, and that the only way to observe a gradation in conditioning to different portions of the CS is to ensure that one portion is preexposed while the other is not. This logic motivated Experiment 3b. All subjects were preexposed to one cue, A. For Group Same presentations of A were either of 10- or 40-s duration, with an equal number of each. Group Different received the same number of preexposure trials, but all stimulus presentations were of 25-s duration, thus equating total duration of exposure in the two groups. In the conditioning phase all rats were conditioned to both A and the novel cue B, with presentations of both stimuli being followed by food after 40 s.

Subjects in Group Different never experienced a stimulus longer than 25 s during the preexposure phase; thus, although the first quarter of A was preexposed on 100% of trials in both groups, the end of the 40-s CS was preexposed in Group Same, but *never* in Group Different—making the difference in preexposure to the first and last quarters of the conditioned duration 100%. We also increased the amount of preexposure from the reduced level given in the previous experiment, to maximize this differential effect. Finally, there was no preexposure to stimulus C in the present experiment, to preclude any generalization of latent inhibition obscuring the results. For example, if subjects in Group Different had been preexposed to C with durations of 10 and 40 s (in parallel with Experiment 3a), the final portion of stimulus C would have been preexposed, even if the final portion of A had not. If this were to generalize more effectively to A, which had also been experienced during the preexposure phase, than to B, which had not, this might minimize the differential latent inhibition between the start and the end of stimulus A at test in Group Different.^5^

### Experiment 3b

#### Method

##### Subjects and apparatus

Subjects were 16 male Lister hooded rats (Charles River, United Kingdom) with a mean free-feeding weight of 405 g (range: 364–436 g). They were housed and food-restricted exactly as in Experiment 1. The apparatus was identical to that of Experiment 1.

##### Procedure

###### Preexposure

All subjects received 10 sessions of preexposure, each comprising 40 presentations of stimulus A. A and B were counterbalanced between click and noise, as in the previous experiments. For Group Same presentations of A had a fixed duration of 40 s on half the trials and 10 s on the remainder; these trials were intermixed in a semirandom order. For subjects in Group Different all CS presentations were of 25-s duration. The ITI comprised a fixed interval of 115 s and a variable interval drawn from an exponential distribution with a mean of 60 s. In all other respects this stage was identical to that in the previous experiments.

###### Conditioning

Conditioning sessions were identical to those of Experiment 1. There were four sessions in this stage.

##### Data treatment

This was identical to that of the previous experiments. The total duration of preexposure of the Target stimulus A was 166 min, considerably more the 66 min of exposure given in Experiment 3a. This might be expected to increase the levels of latent inhibition and thus allow it to be observed at an earlier point in training—and this proved to be the case. Thus the data were analyzed over four sessions in the present experiment.

#### Results

##### Conditioning

**Figure 7 fig7:**
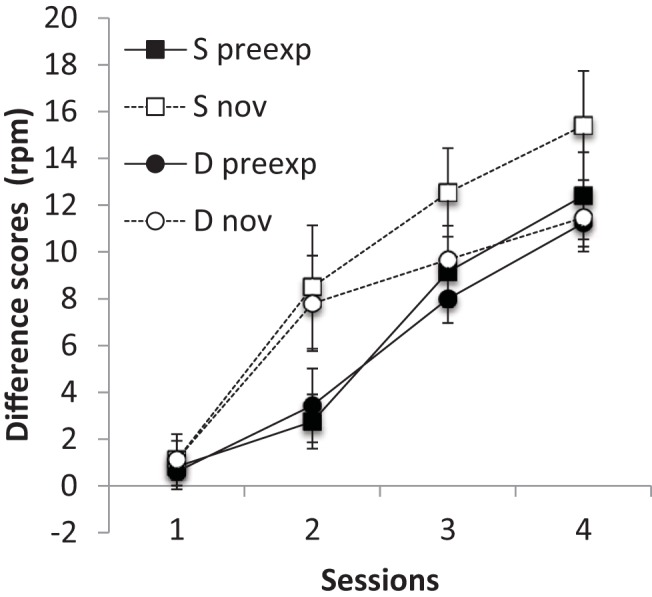
Group mean difference scores (response rate during CS – response rate during pre-CS period) for the preexposed (preexp) and novel (nov) CSs in each of the four conditioning sessions (11–14) of Experiment 3b. Error bars represent standard error of the mean.

The results of the conditioning phase are shown in Figure 7. Robust latent inhibition was apparent in both groups; ANOVA with preexposure (preexposed or not), session and group (Same or Different) as factors revealed a significant effect of session, *F*(3, 42) = 47.10, *p* < .001, *MSe* = 17.45, η_p_^2^ = .77, 95% CI [.61, .83], preexposure *F*(1, 14) = 13.03, *p* = .003, *MSe* = 14.16, η_p_^2^ = .48, 95% CI [.08, .69], and interaction between these two factors, *F*(3, 42) = 6.88 *p* = .001, *MSe* = 4.59, η_p_^2^ = .33, 95% CI [.08, .48]; nothing else was significant, largest *F*(1, 14) = 1.14, *p* = .305, *MSe* = 4.59.[Fig-anchor fig7]

The mean rates of pre-CS responding in each session were 4.16, 8.46, 5.44, and 4.13 rpm for Group Same, and 4.02, 8.10, 4.05, and 3.03 for Group Different. ANOVA with group and sessions as factors revealed only a significant effect of sessions, *F*(3, 42) = 19.53, *p* < .001, *MSe* = 7.41, η_p_^2^ = .58, 95% CI [.34, .69]; nothing else was significant, *F*s < 1.

##### Timing

**Figure 8 fig8:**
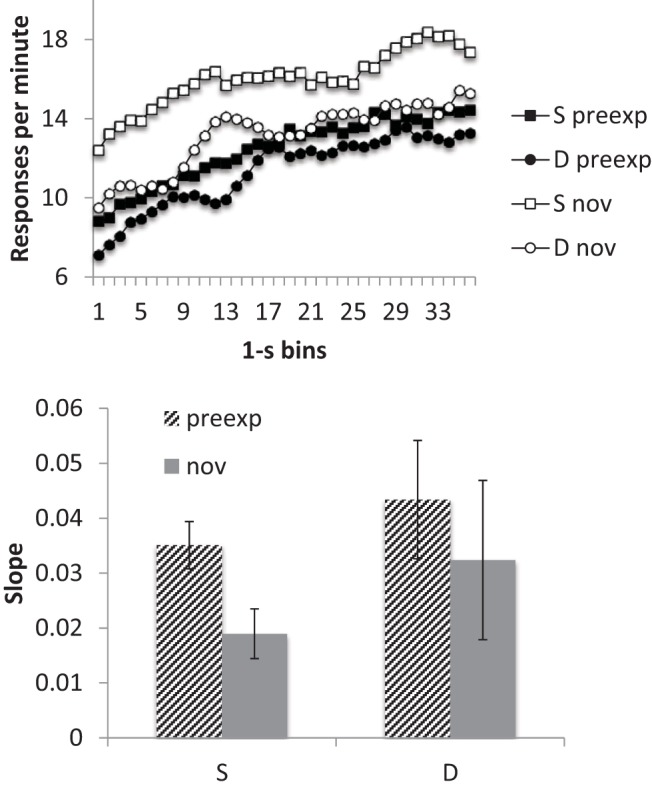
Group mean scores for Group Same (S) and Different (D) for the preexposed (pre) and novel (nov) CSs from the conditioning sessions (11–14) of Experiment 3b. Upper panel: Smoothed response rates in each 1-s bin. Lower panel: Slopes. Bars show standard error of the mean.

The smoothed response rates, averaged over all four sessions, are shown in the upper panel of Figure 8. As in Experiment 3a, there was no obvious sign that conditioning at the end of the CS was greater in Group Different than in Group Same—the difference in responding to preexposed and novel cues appeared greater in Group Same than in Group Different across the entire course of the CS.[Fig-anchor fig8]

The slope data are shown in the lower panel of Figure 8; once again slopes were higher for the preexposed cues: ANOVA with preexposure and group as factors revealed a significant effect of preexposure, *F*(1, 14) = 4.62, *p* = .049, *MSe* = .001, η_p_^2^ = .25, 95% CI [.00, .53]; nothing else was significant, *F*s < 1.

#### Discussion

The results of Experiment 3b mirrored those of Experiment 3a: regardless of whether subjects had experienced the conditioning duration during the preexposure phase, they showed equivalent latent inhibition, and equivalent enhancement in the slope of the preexposed cue’s response function. Despite the greater differential exposure to the start and end portions of the CS in Group Different relative to that in Group Same, there was no sign that this influenced the pattern of results.

We argued above that an associative analysis could explain the enhancement in temporal control after CS preexposure in a number of ways, provided it assumes that the CS can be conceptualised as comprising independent elements that occur in a fixed temporal order, and can suffer latent inhibition and undergo conditioning relatively independently. But if this were the case, one would expect that artificially manipulating the amount of exposure to different portions of the CS could produce differential amounts of latent inhibition, and hence differential levels of conditioning. We could not find any evidence for such an effect, which casts doubt on this assumption.

## General Discussion

Taken together the results from all four experiments supported the assertion that when latent inhibition was obtained, the slope of the preexposed stimulus was higher than that of the novel cue. In Experiments 2, 3 and 4 we found lower levels of responding to the preexposed than to the novel stimulus, and this was accompanied by greater values of slope for the preexposed cue. The results from Experiment 1 were slightly more complex, in that for Group Different there was a latent inhibition effect^6^ but no difference in slope. However, numerically the latent inhibition effect seemed weak in these animals, and analysis of the data after four rather than five training sessions suggested that neither latent inhibition nor an effect of preexposure on slope was present in this group. Taken together these results are consistent with the proposal that the latent inhibition effect produced by stimulus preexposure is accompanied by an increase in temporal control.

**Figure 9 fig9:**
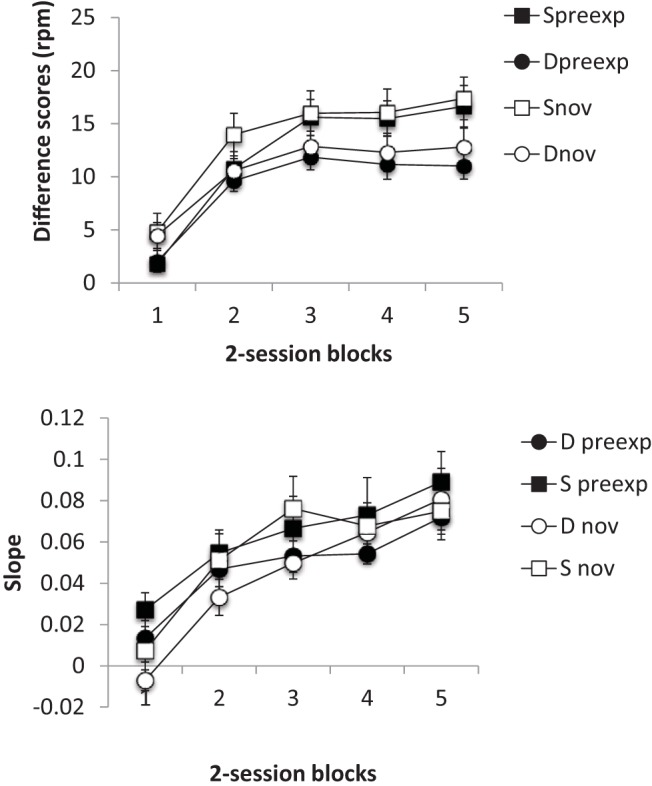
Group mean scores for Group Same (S) and Different (D) for the preexposed (preexp) and novel (nov) CSs from the total, 10 conditioning sessions (11–20) of Experiment 3b. The data are presented in two-session blocks. Group mean difference scores (response rate during CS – response rate during pre-CS period) for the preexposed (preexp) and novel (nov) CSs. Lower panel: Group mean slopes for preexposed and novel cues. Bars show standard error of the mean.

However, one potential reservation with this conclusion relates to the inevitable confound between the slope differences we observed and response rate—in all the experiments we examined slope at a point in training at which latent inhibition was present. This raises the possibility that, because response rates were higher to the novel than to the preexposed CSs, a ceiling effect in responding to the novel stimulus could have been responsible for the shallower slopes observed to this cue. To examine this possibility we conducted six further training sessions in Experiment 3b, and examined how slopes changed in the later parts of training. The conditioning scores and timing slopes were computed as described above, but in five two-session blocks; the resultant data are presented in Figure 9. First, it is evident from the data in the top panel that latent inhibition is most apparent in the first two training blocks; thereafter the main difference seems to be higher rates of responding in Group Same. The slopes, shown in the lower panel, do not show the same pattern, in that they do not appear to asymptote but continue increasing as training progresses. There is also an indication of higher slopes in the preexposed cues, but only in the first two training blocks. The data from the first two blocks correspond to the data that were analyzed above; analysis of the data from the final three blocks is presented here. ANOVA performed on the conditioning scores, with preexposure (preexposed or not), block and group (Same or Different) as factors revealed no significant effects or interactions, largest *F*(1, 14) = 3.75, *p* = .07, *MSe* = 112.28, for the effect of group; neither the effect of block nor any interaction with this factor was significant. This confirms the suggestion that response rates were at asymptote during these final training blocks. A corresponding analysis for the slopes revealed a significant main effect of block, *F*(2, 28) = 5.21, *p* = .012, *MSe* = .001, η_p_^2^ = .27, 95% CI [.02, .47], confirming that slopes were still increasing, and this interacted with preexposure and group, *F*(2, 28) = 6.52, *p* = .005, *MSe* = .001, η_p_^2^ = .32, 95% CI [.04, .51]; nothing else was significant, *Fs* < 1. The interaction was explored further with two Group × Block analysis conducted on the slopes for the preexposed and nonpreexposed cues. For the preexposed cues, this revealed a significant main effect of block, *F*(2, 28) = 8.44, *p* = .001, *MSe* < .001, η_p_^2^ = .38, 95% CI [.08, .56]; nothing else was significant, largest *F*(1, 14) = 1.18, *p* = .30, *MSe* < .003. A corresponding analysis for the nonpreexposed cues revealed nothing significant, largest *F*(2, 28) = 2.31, *p* = .12, *MSe* < .001. In other words, despite the fact that response rates had reached ceiling in both preexposed and nonpreexposed cues, slopes continued to increase, but only for the preexposed stimuli. This is not the pattern that would be predicted if the higher levels of slope in the preexposed cues had been due to a ceiling effect in responding to the novel CS.[Fig-anchor fig9]

Thus the present results suggest that latent inhibition training does not impair temporal control—but that in fact preexposure to a stimulus appeared to *enhance* the ability of subjects to track the time to US delivery. Furthermore, in most cases this was unrelated to the temporal distribution form or duration of the cue that was preexposed—the effect was equally evident regardless of whether or not the subjects are preexposed to the conditioning duration. Finally, we found no evidence that the timing response function was an emergent property of differential latent inhibition to the end of the cue—casting doubt on the suggestion that a CS may be conceptualized as a fixed sequence of independently conditionable elements. Even when the start of the conditioned CS had been preexposed but the end was completely novel—which should foster greater conditioning to the end than to the start of the CS—there was no evidence that this resulted in greater values of slope. It should be noted that we were only able to manipulate exposure to the end of the cue, not to its onset, so it is still logically possible that our effects stemmed from a differential loss of associability at the start of the preexposed stimuli. This is particularly suggested in Experiment 2, for which the smoothed response rates in each 1-s bin of the stimuli appeared lower in the preexposed than the novel cues at the start of training, differences which disappeared in later portions of the stimulus (top panel of Figure 4); however the other experiments did not show such a clear pattern in this regard (top panel of Figures 2, 6 and 8). Moreover, if differential exposure to the start of the CS could bias the response slope function, then one might expect manipulations in exposure to the end of the CS to have a similar effect, and yet we found no evidence of this in Experiment 3. Nonetheless, this possibility must remain as a possible alternative interpretation of our results.

Of course our conclusion of better temporal control relies solely on the slope measure employed in these studies. Although a greater difference in differential responding between the start and end of the CS seems indicative of more accurate timing, it would be far more convincing if we could demonstrate the effect with different measures of timing ability (e.g., the peak procedure). However, obtaining good data from the peak procedure requires a considerable amount of training, and in the current studies this would mean training beyond the point at which latent inhibition had dissipated. Moreover, the peak procedure entails introducing a significant number of longer, nonreinforced trials, which could potentially create a context change, which would also attenuate the latent inhibition effect.

These results do not fit easily within any theoretical framework. The only associative models of conditioning that offer an explanation of timing do so by conceptualizing stimuli in the componential manner we have described above, as (at least in part) a fixed sequence of constituent elements (e.g., Sutton & Barto, 1990; Vogel, Brandon & Wagner, 2003). In principle these theories could explain the enhancement in temporal control after preexposure in terms of differential latent inhibition in different portions of the CS. For example, Vogel et al. (2003) proposed a specific componential way of describing the CS, but the learning rules they employed are based on those of Wagner’s SOP model. According to this account (e.g., Wagner, 1981; cf. McLaren & Mackintosh, 2000), latent inhibition occurs because the CS comes to be predicted by other cues, either those of the experimental context or of the stimulus itself; the stronger these associations the stronger the latent inhibition effect. Moreover, the model can also predict that such associations will form more effectively at the start of the stimulus. Wagner’s SOP theory assumes that initial presentation of the CS leaves the stimulus in an active state that is highly associable, but with time it lapses into a secondary activation state in which it cannot act as a CS or a US in excitatory associations. This would predict that both the experimental context and elements of the stimulus itself will become more strongly associated with the start of the CS than the end, resulting in the start of the CS suffering more latent inhibition. But if such a mechanism were responsible for the enhancement of temporal control produced by preexposure, one should be able to produce such differential latent inhibition artificially, as we attempted in Experiments 3a and 3b. Yet these manipulations appeared to have no effect on the slope of the response function.

The alternative accounts of timing fare no better; for example, although the temporal coding hypothesis (e.g., Barnet, Arnold, & Miller, 1991) can, with added assumptions, explain latent inhibition, it regards timing as a byproduct of conditioning, rather than independent of it. Thus it is hard to see how preexposure, which results in conditioning being less effective in producing the conditioned response, should have the opposite effect on timing. Finally there is also, as we noted above, a large class of time-based models that have been adapted to explain conditioning effects—but it is not clear such accounts can predict latent inhibition, let alone the effect of preexposure on timing. It seems our results present a potential theoretical challenge to theories of both conditioning and timing effects.

In summary, many of the studies that seek to investigate how conditioning and timing interact have focused on learning processes engaged during training that involves reinforcement (e.g., Kirkpatrick & Church, 1998; although see, e.g., Savastano & Miller, 1998). Although this focus has led to a rapid expansion in time-based theories (see Kirkpatrick, 2014 for a recent review), learning phenomena such as latent inhibition, where training occurs in the absence of reinforcement, have tended to be neglected. In this sense the results presented above are completely novel, and also challenging: theoretical models, irrespective of the tradition from which they derive, are unable to adequately account for our findings. Nevertheless, as noted by Kirkpatrick (2014), the speed at which models in the field are evolving suggests that these findings may fuel further theoretical development.

## Figures and Tables

**Table 1 tbl1:** The Design for Each Experiment in this Study

Experiment	Group	Preexposure	Conditioning
1	Same	A (fix 40)	A (fix 40)→food B (fix 40)→food
	Different	A (var 40)	
2	Same	A (fix 20) C (var 20)	A (fix 20)→food B (fix 20)→food
	Different	A (var 20) C (fix 20)	
3a	Same	A (20) C (10, 30)	A (20)→food B (20)→food
	Different	A (10, 30) C (20)	
3b	Same	A (10, 40)	A (40)→food B (40)→food
	Different	A (25)	
*Note*. fix = fixed; var = variable. In all experiments, Group Same was preexposed to the conditioning duration, whereas Group Different was not. In Experiments 1, 2, and 3a, Group Different experienced durations (var in Experiments 1 and 2, fix in Experiment 3a) that were either longer or shorter than, but never the same as, the fixed durations experienced by Group Same. In Experiment 3b, Group Different only experienced durations that were shorter than those experienced by Group Same. Total exposure duration was always identical in the two groups.			

## Footnotes

1 For example, because CS onset initiates a series of memory traces (Machado, 1997), or activity in a series of oscillators with different periods (Church & Broadbent, 1990), or some other time-based process that uniquely defines different portions of the CS (see also e.g., Grossberg & Schmajuk, 1989; Staddon & Higa, 1996).

2 Whereas a control group that is simply placed in the context would, according to this account, learn faster than either of these conditions.

3 We did not use the peak procedure to evaluate timing as latent inhibition is a transient phenomenon, and this method can require a large number of trials to generate sufficient data; latent inhibition is also context-specific, and the introduction of peak trials could produce a context change that would further attenuate the effect.

4 A latent inhibition score was derived for each rat, by subtracting response rates to the preexposed cue from those to the novel cue, summed over all the conditioning sessions. This score was more than two standard deviations below the mean for this animal.

5 This strategy introduced the risk of there being a greater context change between preexposure and test for Group Different, which had never experienced a 40-s stimulus before the conditioning phase, than in Group Same, which had. But conversely subjects in Group Same experienced two stimulus durations during preexposure, but only one at test—meaning that in this respect it underwent a greater context change between preexposure and test than subjects in Group Different, which experienced only one stimulus duration throughout. It was hoped that these effects would offset each other, and roughly equate the similarity between preexposure and test contexts in the two groups.

6 More precisely, there was a Preexposure × Sessions interaction that did not interact with group; we did not examine latent inhibition in Group Different separately.
